# Outcomes of patients admitted to intensive care units for acute manifestation of small-vessel vasculitis: a multicenter, retrospective study

**DOI:** 10.1186/s13054-016-1189-5

**Published:** 2016-01-26

**Authors:** Antoine Kimmoun, Elisabeth Baux, Vincent Das, Nicolas Terzi, Patrice Talec, Pierre Asfar, Stephan Ehrmann, Guillaume Geri, Steven Grange, Nadia Anguel, Alexandre Demoule, Anne Sophie Moreau, Elie Azoulay, Jean-Pierre Quenot, Julie Boisramé-Helms, Guillaume Louis, Romain Sonneville, Nicolas Girerd, Nicolas Ducrocq, Nelly Agrinier, Denis Wahl, Xavier Puéchal, Bruno Levy

**Affiliations:** 1 Brabois Medical Intensive Care Unit, Nancy University Hospital, Vandoeuvre-les-Nancy, Nancy, 54000 France; 2 INSERM U1116, Vandoeuvre-les-Nancy, Nancy, France; 3 Medical-Surgical Intensive Care Unit, Andre Gregoire District Hospital Center, Montreuil, F-93105 France; 4 Medical Intensive Care Unit, Caen University Hospital, Avenue de la Côte de Nacre, 14000 Caen, France; 5 Medical Intensive Care Unit, Angers University Hospital, Angers, F-49933 France; 6 Medical Intensive Care Unit, Bretonneau University Hospital, Tours, F-37044 France; 7 Medical Intensive Care Unit, Cochin University Hospital, Paris, F-75014 France; 8 Medical Intensive Care Unit, Rouen University Hospital, Rouen, 76031 France; 9 Medical Intensive Care Unit, Kremlin-Bicêtre University Hospital, Paris, F-94275 France; 10 Medical Intensive Care Unit and Respiratory Division, Pitié-Salpêtrière University Hospital, Paris, 75013 France; 11 Medical-Surgical Intensive Care Unit, Lille University Hospital, Lille, F-59000 France; 12 Medical Intensive Care Unit, Saint-Louis University Hospital, Paris, 75010 France; 13 Medical Intensive Care Unit, Dijon University Hospital, Dijon, F-21079 France; 14 Medical Intensive Care Unit, NHC University Hospital, Strasbourg, F-67091 France; 15 Medical Intensive Care Unit, Mercy Regional Hospital, Ars-Laquenexy, 57530 France; 16 Medical Intensive Care Unit, Bichat - Claude-Bernard University Hospital, Paris, 75018 France; 17 INSERM CIC1433, Nancy University Hospital, Nancy, 54000 France; 18 INSERM CIC-EC, CIE6, Nancy University Hospital, Nancy, 54000 France; 19 Vascular Medicine Division and Regional Competence Center for Rare Vascular and Systemic Autoimmune Diseases, Nancy University Hospital, Vandoeuvre-les Nancy, Nancy, 54511 France; 20 National Referral Center for Necrotizing Vasculitides and Systemic Sclerosis, Cochin Hospital, University Paris Descartes, Paris, F-75014 France

**Keywords:** Intensive care unit, Small-vessel vasculitis, Outcome

## Abstract

**Background:**

The outcomes of patients admitted to the intensive care unit (ICU) for acute manifestation of small-vessel vasculitis are poorly reported. The aim of the present study was to determine the mortality rate and prognostic factors of patients admitted to the ICU for acute small-vessel vasculitis.

**Methods:**

This retrospective, multicenter study was conducted from January 2001 to December 2014 in 20 ICUs in France. Patients were identified from computerized registers of each hospital using the International Classification of Diseases, Ninth Revision (ICD-9). Inclusion criteria were (1) known or highly suspected granulomatosis with polyangiitis, eosinophilic granulomatosis with polyangiitis, microscopic polyangiitis (respectively, ICD-9 codes M31.3, M30.1, and M31.7), or anti–glomerular basement membrane antibody disease (ICD-9 codes N08.5X-005 or M31.0+); (2) admission to the ICU for the management of an acute manifestation of vasculitis; and (3) administration of a cyclophosphamide pulse in the ICU or within 48 h before admission to the ICU. The primary endpoint was assessment of mortality rate 90 days after admission to the ICU.

**Results:**

Eighty-two patients at 20 centers were included, 94 % of whom had a recent (<6 months) diagnosis of small-vessel vasculitis. Forty-four patients (54 %) had granulomatosis with polyangiitis. The main reasons for admission were respiratory failure (34 %) and pulmonary-renal syndrome (33 %). Mechanical ventilation was required in 51 % of patients, catecholamines in 31 %, and renal replacement therapy in 71 %. Overall mortality at 90 days was 18 % and the mortality in ICU was 16 %. The main causes of death in the ICU were disease flare in 69 % and infection in 31 %. In univariable analysis, relevant factors associated with death in nonsurvivors compared with survivors were Simplified Acute Physiology Score II (median [interquartile range] 51 [38–82] vs. 36 [27–42], *p* = 0.005), age (67 years [62–74] vs. 58 years [40–68], *p* < 0.003), Sequential Organ Failure Assessment score on the day of cyclophosphamide administration (11 [6–12] vs. 6 [3–7], *p* = 0.0004), and delayed administration of cyclophosphamide (5 days [3–14] vs. 2 days [1–5], *p* = 0.0053).

**Conclusions:**

Patients admitted to the ICU for management of acute small-vessel vasculitis benefit from early, aggressive intensive care treatment, associated with an 18 % death rate at 90 days.

**Electronic supplementary material:**

The online version of this article (doi:10.1186/s13054-016-1189-5) contains supplementary material, which is available to authorized users.

## Background

The revised International Chapel Hill Consensus Conference Nomenclature of Vasculitides [1] characterizes vasculitis as a function of the size of the vessel involved. According to this nomenclature, small-vessel vasculitides (SVV) are a group of diseases that includes antineutrophil cytoplasmic antibody–associated vasculitis (AAV) and immune complex SVV. Epidemiological data on SVV remain scarce, although they could be considered as orphan diseases [2]. With the development of therapeutic strategies that include corticosteroids, immunosuppressants, and (plasma exchange [PLEX]), SVV survival rates have considerably improved, from 30 to 75 % at 5 years [3]. The leading causes of death are related mainly to a life-threatening disease at the time of diagnosis or long-term complications of immunosuppressive therapies, all of which may require intensive care unit (ICU) admission [4, 5]. To date, there are only few studies, all retrospective, in which researchers have reported the outcomes of patients with vasculitis admitted to the ICU. The reported ICU mortality rates of the three most recent studies ranged from 11 to 52 % [6–8]. AAV, and particularly granulomatosis with polyangiitis (GPA; Wegener’s granulomatosis), was the most frequent form of vasculitis. Unfortunately, all three were single-center studies and heterogeneous in nature, as they included several types of vasculitides with different prognoses. Patients were admitted either in the initial phase of the disease or after a long-term evolution. Finally, therapeutic vasculitis management was poorly described and mostly inhomogeneous between studies.

Nonspecific ICU scores at admission, such as the Simplified Acute Physiology Score II (SAPS II) or Sequential Organ Failure Assessment (SOFA) score, have been reported to be associated with outcome; however, specific vasculitis scores are not adapted to the ICU setting. In light of these circumstances, we carried out a retrospective, multicenter study to describe the clinical course, outcomes, and prognostic factors of patients admitted to the ICU for acute manifestation of new-onset SVV.

## Methods

### Study design

In this retrospective, observational, multicenter study, 22 ICUs in northern France were contacted individually by e-mail on three occasions to analyze the outcomes of patients admitted to the ICU for acute manifestations of SVV. Two of the centers did not partake in the study. In the 20 participating centers, patients were identified by two methods:

1. We used the computerized registers of each hospital to identify patients with the International Classification of Diseases, Ninth Revision (ICD-9), codes M31.3 for GPA, M30.1 for eosinophilic GPA, M31.7 for microscopic polyangiitis, and N08.5X-005 or M31.0+ for anti–glomerular basement membrane (GBM) antibody disease.
2. If no patient was found in the computerized database of the medical informatics department, then the following keywords were searched in the hospital report database of each ICU department: “microscopic polyarteritis,” “granulomatosis with polyangiitis (or Wegener’s),” “eosinophilic granulomatosis with polyangiitis (or Churg-Strauss),” “anti-glomerular basement membrane disease (or Goodpasture syndrome).”

All patients admitted to the ICU for SVV management were screened. When a patient was hospitalized in the ICU on more than one occasion, only the first ICU admission was considered.

### Inclusion criteria

To be included, patients had to fulfill the following criteria:

1. Patients had to be admitted to the ICU for acute manifestations of known or highly suspected SVV (new diagnosis or relapse). On the basis of the results of previously published studies, acute manifestations of known or highly suspected SVV requiring admission in ICU include respiratory failure, acute renal failure, cardiac failure, coma due to central nervous system involvement, and severe gastrointestinal involvement (e.g., peritonitis due to small intestine perforation) [6, 9, 10].
2. Patients had to receive cyclophosphamide pulse therapy according to French recommendations [11, 12] within 48 h before admission or during their ICU stay.
3. Primary SVV patients were included if they presented with a diagnosis of AAV: microscopic polyarteritis, GPA (formerly known as Wegener’s granulomatosis), and eosinophilic GPA (formerly known as Churg–Strauss syndrome). Due to similar clinical presentation and initial treatment in the ICU, patients with anti–GBM antibody disease (an immune complex vasculitis formerly known as Goodpasture syndrome) were also included in this study.

### Exclusion criteria

Considering their heterogeneous clinical presentation and management, other immune complex SVV (cryoglobulinemic vasculitis, immunoglobulin A vasculitis, hypocomplementemic urticarial vasculitis [anti-C1q vasculitis]) were excluded from this analysis. Details on SVV not included in the present study are provided in Additional file 1. Patients admitted for an infectious complication secondary to SVV immunosuppressive treatments were excluded from the study.

### Data collection

Each clinical record, in either paper or electronic form, was reviewed at each site by the principal investigator. All scores were calculated by the same principal investigator to ensure interscore reliability. At ICU admission, the following data were collected for each patient: demographic data; reason for admission; medical history; SVV diagnosis type; and disease assessment scores, including SAPS II score, SOFA score, Birmingham Vasculitis Activity Score (BVAS) (version 3), and revised Five-Factor Score (FFS).

SAPS II and SOFA scores were used to assess disease severity. The SAPS II score is calculated using the worst 12 physiological variables during the first 24 h in the ICU and also includes three disease-related variables [13]. The SOFA score is based on six physiological variables and can be calculated on a daily basis [14].

Vasculitis disease activity was assessed on the basis of the BVAS [15]. This score is based on clinical and biological items in nine separate organ systems: general; cutaneous; mucous membrane and eyes; ear, nose, and throat; cardiovascular; gastrointestinal; pulmonary; renal; and nervous system. The revised FFS was calculated at admission for patients with microscopic polyangiitis, GPA, eosinophilic granulomatosis with polyangiitis, and anti–GBM antibody disease. This score is used to assess prognosis at the time of diagnosis and includes the following items: serum creatinine level (>150 μmol or <150 μmol); presence of severe gastrointestinal tract involvement; cardiomyopathy; age; and ear, nose, and throat involvement [10].

### Study endpoints

The primary endpoint was assessment of mortality rate 90 days after ICU admission. Outcome was also recorded (survivors and nonsurvivors) in the ICU and at day 90. For each patient, three specific adverse events reflecting global consequences of immunosuppression were recorded during the ICU stay: sepsis, hemorrhagic syndrome, and hematological disorders such as aplasia and thrombopenia. The incidence of these adverse events was collected only if they occurred at least 48 h after the cyclophosphamide pulse. The duration between the cyclophosphamide pulse and each adverse event was also recorded. Details are provided in Additional file 1.

### Ethics

According to French law (L.1121-1 paragraph 1 and R1121-2, Public Health Code), neither informed consent nor approval of an ethics committee was necessary for anonymous data extraction from and analysis of patients’ medical files.

### Statistical analysis

Continuous variables are presented as median and interquartile range, and categorical variables are reported as frequency (percent). Two groups were defined according to 90-day mortality: survivors and nonsurvivors. Comparison between the two groups was performed on continuous variables using Mann–Whitney *U* tests due to a nonnormal distribution of all variables. For qualitative variables, a *χ* ^2^ test or Fisher’s exact test was used as appropriate. Correlations were assessed using the Pearson correlation test. Association between baseline ICU characteristics with mortality was assessed in univariable and multivariable logistic regression. Given the low number of events, only two explanatory variables could be entered in the multivariable models; that is, several models were constructed, each containing two explanatory variables. These candidate variables entered in multivariable analysis were chosen on the basis of the preceding univariable analysis (entry criteria *p* < 0.05 in univariable analysis). Models adjusted for various possible confounders (age, SOFA score at admission, SAPS II at admission) were ultimately presented. Because of the absence of a universally accepted threshold, continuous variables were categorized according to the thresholds identified using the Youden index from receiver operating characteristic curve analyses. Mortality was described using Kaplan–Meier survival estimates and compared between the group baseline characteristics by log-rank tests. All analyses were performed using Prism software (GraphPad Software, La Jolla, CA, USA) and IBM SPSS Statistics 20.0 software (IBM, Armonk, NY, USA). The two-tailed significance level was set at *p* < 0.05.

## Results

### Population characteristics

**Fig. 1:**
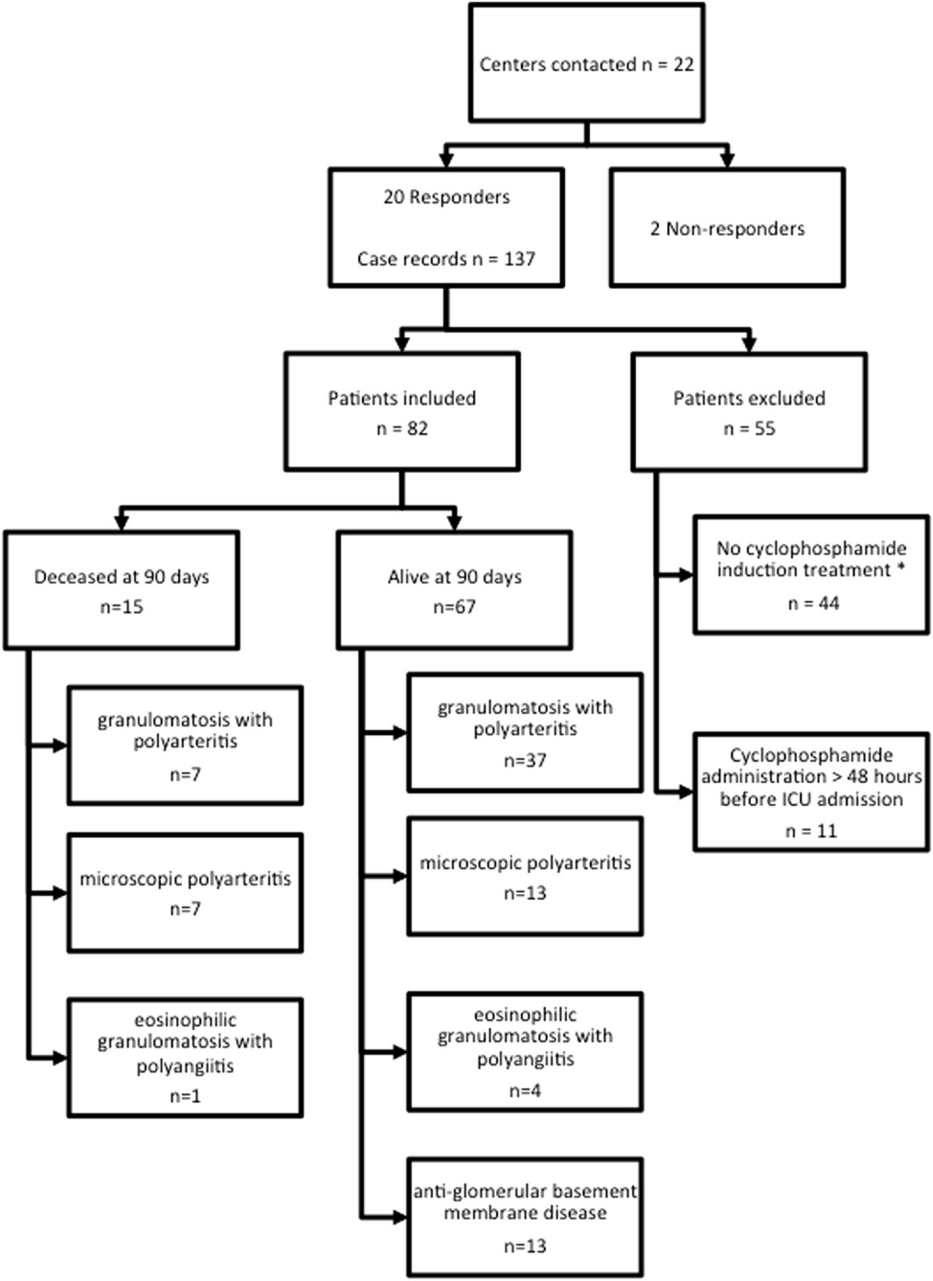
Flowchart of the included patients with outcome at 90 days, * no patient received rituximab

The study population characteristics are provided in Table 1. In the 20 participating centers, 82 patients (36 women, 46 men) with a median age of 67.0 years (63.0–74.5) were included from January 2001 to December 2014 (Fig. 1). The delay between traditional hospitalization wards and ICU admission was 6.5 days (1–14). Among the study population, 52 patients (63 %) had no prior medical history and 78 patients (95 %) had a performance status score of 0 or 1. Of the included patients, 77 (94 %) were admitted for a new or recent diagnosis of SVV, with GPA (Wegener’s) being the main diagnosis (44 patients, 54 %). Thirteen patients (16 %) were admitted to the ICU for an anti-GBM antibody disease. The predominant clinical patterns at admission were pulmonary-renal syndrome (27 patients, 33 %), isolated respiratory failure (28 patients, 34 %), and isolated renal failure (24 patients, 29 %). Reasons for admission for all patients with acute renal failure were indications of renal replacement therapy with the need to pursue PLEX. SAPS II and BVAS at admission were 37.5 (28.0–46.5) and 16.0 (12.0–20.0), respectively.

**Table 1:** Baseline demographic characteristics of 82 study patients at admission to ICU

Characteristics	Data
Age, yr	67.0 (63.0–74.5)
Female sex	36 (44)
Medical history	
Malignant disease	5 (6)
Chronic renal failure	7 (8)
Heart failure	11 (13)
Chronic respiratory failure	7 (8)
Neurological failure	1 (1)
Diabetes	5 (6)
Malnutrition	2 (2)
None	52 (63)
Performance status^a^	
0: Normal activity	37 (45)
1: Symptomatic but completely ambulatory	41 (50)
2: Less than 50 % of daytime in bed	4 (5)
3: More than 50 % of daytime in bed	0 (0)
4: Totally confined to bed or chair	0 (0)
Small-vessel vasculitis diseases	
Granulomatosis with polyangiitis	44 (54)
Microscopic polyangiitis	20 (24)
Eosinophilic granulomatosis with polyangiitis	5 (6)
Anti–glomerular basement membrane antibody disease	13 (16)
Disease status	
Newly or recently diagnosed	77 (94)
Relapsing disease	5 (6)
Patient receiving chronic immunosuppressive therapy^b^	4 (5)
Cause of admission	
Respiratory failure	28 (34)
Acute renal failure	24 (29)
Pulmonary-renal failure	27 (33)
Septic shock	1 (1)
Others^c^	3 (4)
Disease and severity assessment scores at admission	
Simplified Acute Physiology Score II	37.5 (28.0–46.5)
Sequential Organ Failure Assessment score	5.0 (4.0–8.0)
Birmingham Vasculitis Activity Score	16.0 (12.0–20.0)
Revised Five-Factor Score	2.0 (1.0–2.0)

Data are presented as number (%) or median (interquartile range)

^a^Missing data: 3

^b^Missing data: 1

^c^Two patients with encephalitis and one with myocarditis

### Small-vessel vasculitis and ICU management

Data for small-vessel vasculitis and ICU management are provided in Table 2. All patients received cyclophosphamide with a median dose of 1000 mg (800–1000). Glucocorticoid pulses were administered in 74 patients (90 %), and 79 patients (96 %) received daily high-dose glucocorticoids. PLEX was performed in 63 patients (77 %). In the ICU, 42 patients (51 %) required mechanical ventilation during 11.5 days (8.0–22.5) and 25 patients (31 %) received vasopressor therapy during 7.0 days (3.0–18.5). Renal replacement therapy was performed in 58 patients (71 %) for 13.0 days (8.0–20.75) and was maintained after ICU stay in 28 patients (34 %).

**Table 2:** Small-vessel vasculitis and intensive care management

	Data (*N* = 82 patients)
Small-vessel vasculitis management	
Number of patients receiving glucocorticoid induction treatment	74 (90)
Number of days	3.0 (3.0–3.0)
Total dose, mg methylprednisolone equivalents	1500 (1500–3000)
Number of patients receiving daily glucocorticoids after induction treatment	79 (96)
Number of patients receiving plasma exchange	63 (77)
Number of sessions	7.0 (5.0–7.0)
Number of patients receiving cyclophosphamide pulse	82 (100)
Induction dose, mg	1000 (800–1000)
Number of patients receiving rituximab	3 (4)
ICU management	
Number of patients receiving mechanical ventilation^a^	42 (51)
Duration of mechanical ventilation, days	11.5 (8.0–22.5)
Number of patients receiving venovenous extracorporeal membrane oxygenation	6 (7)
Number of patients receiving catecholamines	25 (31)
Duration of catecholamine administration, days	7.0 (3.0–18.5)
Number of patients receiving renal replacement therapy in ICU	58 (71)
Duration of renal replacement therapy in ICU, days	13.0 (8.0–20.75)
Number of patients receiving renal replacement therapy before ICU stay	11 (13)
Number of patients receiving renal replacement therapy after ICU stay	28 (34)

*ICU* intensive care unit

Data are presented as number (%) or median (interquartile range)

^a^Including invasive and noninvasive ventilation

### Adverse events in the ICU

Data for adverse events in the ICU are given in Table 3. Nine patients (11 %) presented with neutropenia <1500/mm^3^ after the cyclophosphamide pulse, three (4 %) of whom had a nadir <500/mm^3^. Infection was reported in 25 patients (30 %), with the lung being the most frequently infected site (15 patients, 60 %), predominantly by Gram-negative microorganisms (16 patients, 64 %). Unfavorable evolution toward septic shock was observed in 13 patients (16 %). Venovenous extracorporeal membrane oxygenation was initiated for refractory respiratory failure in six patients (7 %), four of whom survived. Lastly, 57 patients (69 %) presented with at least one hemorrhagic syndrome during their ICU stay. The main cause of death in the ICU was disease flare in 69 % of cases, followed by infection in 31 % of cases.

**Table 3:** Summary of prespecified adverse events recorded in the ICU

	Data (*N* = 82 patients)
Neutropenia^a^ <1500/mm^3^	9 (11)
Delay between cyclophosphamide administration and neutropenia^a^ <500/mm^3^, days	16 (2–25)
Number of patients with infection	25 (30)
Location	
Urinary tract	2 (8)
Lung	15 (60)
Bacteremia	4 (16)
Others	4 (16)
Bacterial source	
Gram-positive	3 (12)
Gram-negative	16 (64)
Other^b^	1 (4)
No pathogen identified	5 (20)
Delay between ICU admission and first infection event, days	13.0 (4.5–19.75)
Number of patients with septic shock	13 (16)
Number of patients presenting with hemorrhagic syndrome	57 (69)
Number of packed red blood cells infused during ICU stay	4.0 (0–7.5)
Delay between ICU admission and first hemorrhagic event, days	1.0 (0–5.0)
Cause of death in ICU	
Infection	4 (31)
Disease flare	9 (69)

*ICU* intensive care unit

Data are presented as number (%) or median (interquartile range)

^a^Missing data: 2

^b^Virus

### Comparison between survivors and nonsurvivors at 90 days

Data derived from comparison of survivors and nonsurvivors at 90 days are provided in Table 4, and the results of univariable and multivariable analyses are given in Table 5.

**Table 4:** Comparison of survivors and nonsurvivors at 90 days

	Survivors (*n* = 67)	Nonsurvivors (*n* = 15)	*p* Value
Age, yr	58.0 (40.0–68.0)	67.0 (62.0–74.0)	0.003
Female sex	30 (44)	6 (40)	0.78
Medical history			
Malignant disease	3 (4)	2 (13)	0.055
Chronic renal failure	6 (9)	1 (6)	
Chronic respiratory failure	5 (7)	2 (13)	
Heart failure	6 (9)	5 (33)	
Neurological failure	1 (1)	0 (0)	
Diabetes	3 (4)	2 (13)	
Malnutrition	1 (1)	1 (6)	
None	46 (68)	6 (40)	
Performance status^a^	2.0 (1.0–2.0)	2.0 (1.0–2.0)	0.68
Small-vessel vasculitis diseases			
Granulomatosis with polyangiitis	37 (55)	7 (47)	0.06
Microscopic polyangiitis	13 (20)	7 (47)	
Eosinophilic granulomatosis with polyangiitis	4 (5)	1 (6)	
Anti–glomerular basement membrane antibody disease	13 (20)	0 (0)	
Delay between hospitalization ward to admission to ICU, days	5.0 (1.0–12.0)	11.0 (2.0–28.0)	0.21
Reason for admission			
Respiratory failure	1 (29)	8 (54)	0.37
Acute renal failure	22 (32)	2 (13)	
Pulmonary-renal failure	22 (32)	5 (33)	
Septic shock	1 (2)	0 (0)	
Others^b^	3 (5)	0 (0)	
Number of patients receiving glucocorticoid induction treatment	62 (92)	12 (80)	0.15
Number of patients receiving plasma exchange	50 (75)	13 (86)	0.50
Disease and severity assessment scores at admission			
Revised Five-Factor Score	2.0 (1.0–2.0)	2.0 (1.0–3.0)	0.88
Simplified Acute Physiology Score II	36 (27–42)	51.0 (38.0–82.0)	0.005
Sequential Organ Failure Assessment score at admission	4.0 (4.0–7.0)	8.0 (6.0–9.0)	0.008
Birmingham Vasculitis Activity Score	16.0 (12.0–20.0)	16.0 (12.0–20.0)	0.85
Sequential Organ Failure Assessment score at cyclophosphamide administration	6.0 (3.0–7.0)	11.0 (6.0–12.0)	0.0004
Delay between ICU admission and cyclophosphamide administration, days	2.0 (1.0–5.0)	5.0 (3.0–14.0)	0.0053

*ICU* intensive care unit

Data are presented as number (%) or median (interquartile range)

^a^Missing data: 3

^b^Two patients with encephalitis and one with myocarditis

**Table 5:** Results of uni- and multivariable analysis

	Association between delay from ICU admission to cyclophosphamide administration and outcome		Association between SOFA score at cyclophosphamide administration and outcome	
Model	OR (CI)	*p*	OR (CI)	*p*
Univariable model	1.15 (1.04–1.28)	0.007	1.32 (1.13–1.55)	<0.001
Multivariable models				
Adjusted for age	1.14 (1.03–1.27)	0.01	1.33 (1.12–1.58)	0.001
Adjusted for SOFA score at admission	1.16 (1.04–1.28)	0.008	1.35 (1.11–1.64)	0.003
Adjusted for SAPS II at admission	1.16 (1.04–1.29)	0.01	1.20 (0.96–1.48)	0.11
Adjusted for SOFA score at cyclophosphamide administration	1.16 (1.05–1.29)	0.005	–	–
Adjusted for delay between ICU admission and cyclophosphamide administration	–	–	1.35 (1.14–1.60)	<0.001

*CI* confidence interval, *ICU* intensive care unit, *OR* odds ratio, *SAPS* Simplified Acute Physiology Score, *SOFA* Sequential Organ Failure Assessment

Data are presented as odds ratio (95 % confidence interval)

**Fig. 2:**
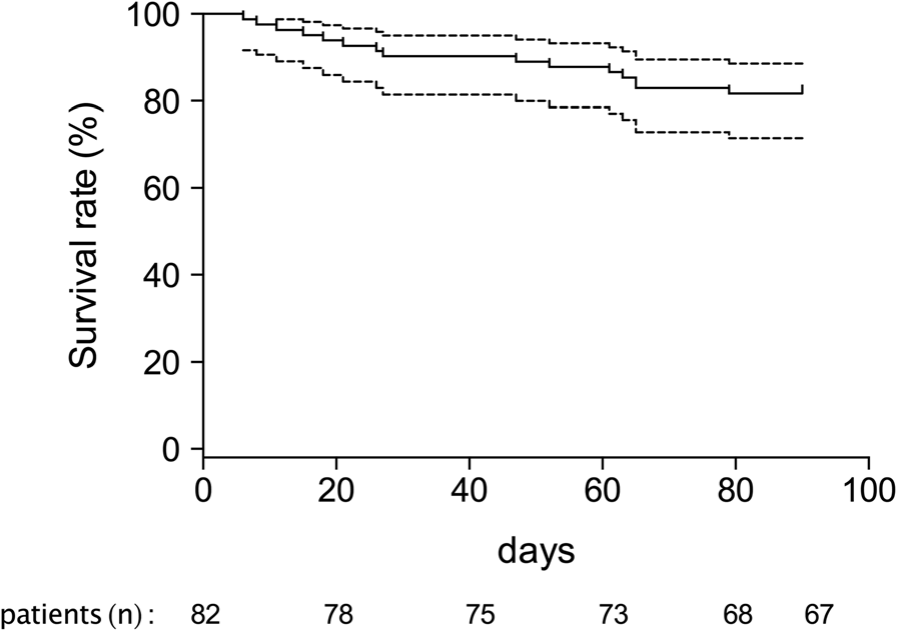
Kaplan–Meyer curves estimating the rate of survival at 90 days. The *dashed line* represents the 95 % confidence interval. Values below each time point indicate the number of surviving patients

Overall mortality was 18 % (15 deaths) (Fig. 2). All patients with an anti-GBM disease survived at 90 days. Anti-GBM disease is known to have a better prognosis, which may have lowered the mortality rate. After removing patients with anti-GBM disease and considering only patients with AAV, we found that the mortality rate in the ICU and at 90 days remained less than 20 % and less than 25 %, respectively.

Sex, medical history, performance status before ICU admission, vasculitis type, delay between hospitalization ward and ICU admission, reason for admission, induction treatment for SVV, revised FFS, BVAS, and SOFA score at admission were not significantly different between survivors and nonsurvivors.

**Fig. 3:**
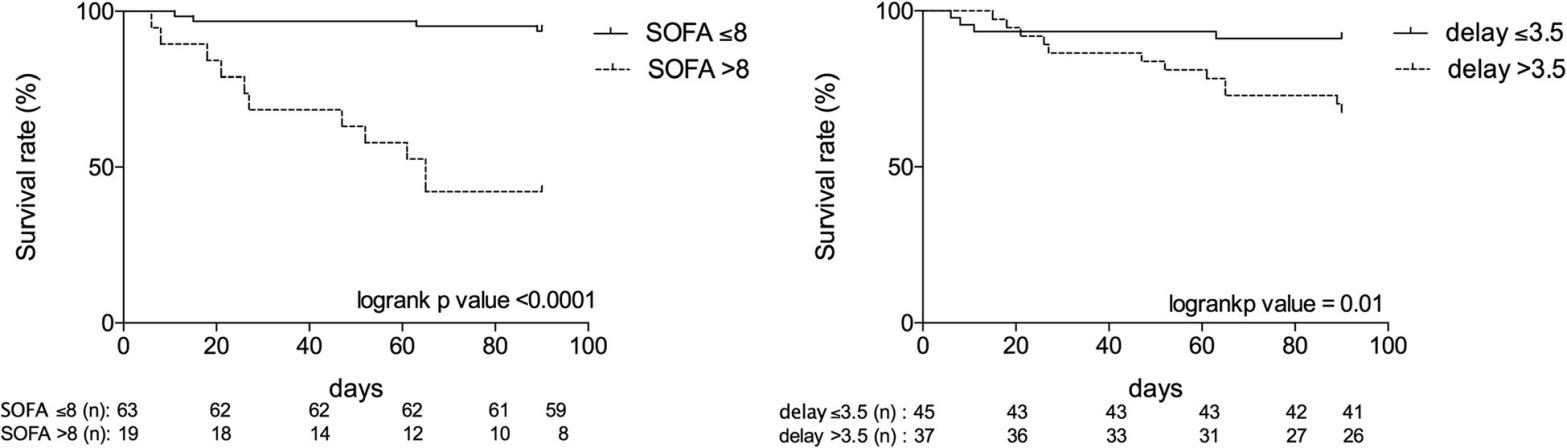
Kaplan–Meyer curves estimating the rate of survival for a Sequential Organ Failure Assessment (SOFA) score >8 on the day of cyclophosphamide administration (*left panel*) and for a delay in cyclophosphamide administration >3.5 days (*right panel*). Values below each time point indicate the number of surviving patients

Nonsurvivors were older than survivors (67 years [62.0–74] vs. 58.0 years [40–68], *p* = 0.003). SAPS II score was also significantly higher at ICU admission in nonsurvivors than in survivors (51 [38–82] vs. 36 [27–42], *p* = 0.005). A higher SOFA score on the day of cyclophosphamide administration (survivors 6 [3–7] vs. nonsurvivors 11 [6–12], *p* = 0.0004), with a threshold value of 8 (sensitivity 73 %, specificity 88 %), was associated with death (Fig. 3). A delayed administration of cyclophosphamide after ICU admission (survivors 2.0 days [1.0–5.0] vs. nonsurvivors 5.0 days [3.0–14.0], *p* = 0.0053), with a threshold value of 3.5 days (sensitivity 73 %, specificity 61 %), was also associated with unfavorable evolution.

In univariable logistic regression, SOFA score on the day of cyclophosphamide administration and timing between admission and administration of cyclophosphamide were significantly associated with outcome (respectively, odds ratio [OR] with 95 % confidence interval [CI] for a 1-point increase in SOFA score 1.32 [1.13–1.55], *p* < 0.001; and for a 1-day increase in delay 1.15 [1.04–1.28]; *p* = 0.007) (Table 5).

In multivariable analysis (Table 5), both SOFA score on the day of cyclophosphamide administration and timing between admission and administration of cyclophosphamide were significantly associated with outcome (OR for a 1-day increase in delay 1.16 [95 % CI 1.05–1.29], *p* = 0.005; and OR for a 1-point increase in SOFA 1.35 [1.14–1.60]; *p* < 0.001). All other models identified a significant association for delay between admission and either administration of cyclophosphamide or SOFA score on the day of cyclophosphamide administration, except when adjusted for SAPS II (OR 1.20 [95 % CI 0.96–1.48], *p* = 0.11).

All nonsurvivors received mechanical ventilation and vasopressor therapy (Additional file 2: Table 6).

## Discussion

The main results of the present multicenter study of patients admitted to the ICU with SVV are as follows: (1) mortality represented about one-fifth of the included population, despite life-threatening manifestations at admission requiring aggressive immunosuppressive therapy; (2) subject to other undetected confounding factors that we were not able to include in the multivariable analysis, ICU severity score, such as SOFA score on the day of cyclophosphamide administration in the ICU, also seemed to be associated with unfavorable outcome; and (3) delayed administration of cyclophosphamide was also likely associated with death.

### Causes of ICU admission

Due to the noninclusion criteria, only one patient presented with septic shock at admission and was diagnosed thereafter with AAV. Consequently, all patients were admitted for acute manifestations of the disease, which consisted mainly of acute respiratory failure or/and acute renal failure. Overall, respiratory failure was present in two-thirds of our patients. In accordance with this, in the studies of Khan et al. [7] and Monti et al. [16], clinical presentations such as acute respiratory failure related to diffuse intraalveolar hemorrhage were also reported to be the first manifestation of AAV at ICU admission.

### Immunosuppressive therapy–related infection in the ICU

The rate of acquired infection hovered at 30 % and was surprisingly less than that of other populations usually admitted to the ICU [17]. This low rate of infection may be explained by the low exposure of patients to chronic immunosuppressive therapies: Only four patients had been receiving chronic immunosuppressive therapy for more than 6 months before ICU admission. In a retrospective series, Cruz et al. found that patients admitted for an infectious process tended to have a higher mortality rate [9]. Similarly, Befort et al. recently reported that cause of death was related mainly to an infectious process in 61 % of ICU patients [6]. Prolonged exposure to immunosuppressive therapies such as corticosteroids before ICU admission is also known to be independently associated with a higher risk of death [18]. Conversely, results from the CORTAGE trial confirmed that low cumulative doses of corticosteroids and limited doses of cyclophosphamide at 500 mg per pulse were associated with a lower occurrence of infection in the elderly [19].

Cyclophosphamide has long been the standard induction treatment in acute manifestations of severe AAV. Randomized controlled trials have also shown that rituximab was noninferior to cyclophosphamide therapy for remission induction in these patients [20, 21]. However, the latter study excluded patients with either alveolar hemorrhage sufficiently severe to require mechanical ventilation or with a serum creatinine level greater than 350 μmol/L. Patient subset analyses including one-fourth of participants with diffuse alveolar hemorrhage or those with major renal disease did not reveal any between-arm differences in remission rate [21]. In these studies, there were no significant differences between the two treatments with respect to adverse events. In the particular setting of the ICU, one can speculate whether rituximab would not be safer than cyclophosphamide for infectious adverse events [22].

It is noteworthy that a high number of our patients were treated with PLEX as an adjunct for frequent acute respiratory failure and/or acute renal failure at patient admission. Patients with respiratory failure due to diffuse alveolar hemorrhage are thought to benefit from PLEX, and the rate of renal recovery in AAV presenting with renal failure has furthermore been shown to be increased with PLEX [23]. The latter is the subject of a large, ongoing, multicenter randomized controlled trial to confirm these data in this patient population (PEXIVAS; ClinicalTrials.gov identifier NCT00987389).

### Prognostic factors

Despite increased use, intensivists do not routinely prescribe immunosuppressive therapies for the management of severe vasculitis. In the ICU, their prescription in instances of multiple organ failure could seem counterintuitive at first glance and most often is associated with an increased complication rate and potentially with a negative outcome. In view of our results, this paradigm appears not to be justified for acute manifestations of SVV. Previous studies have furthermore found highly heterogeneous results with regard to ICU mortality (11–52 %). One major reason may be related to the heterogeneity of the included population. Indeed, most of these studies involved, on one hand, mixed samples including relapse and new diagnoses of various classes of necrotizing vasculitis and, on the other hand, acute manifestations of the disease as well as chronic immunosuppressive-related infections [6, 7, 9, 18, 24]. Owing to the high degree of homogeneity of our population, only a small number of factors appeared to be associated with ICU mortality. As expected, a high  SAPS II score, which is a nonspecific ICU severity score assessed at admission, was associated with worst outcome. This score was also systematically found to be predictive of ICU mortality in all other previous studies [6, 7, 9, 18, 25]. In univariable and multivariable analysis, SOFA score measured on the day of cyclophosphamide administration in the ICU was strongly associated with a poorer outcome. The delayed administration of cyclophosphamide in the ICU was also associated with a higher mortality rate. Considering that all patients included in this study presented with an acute manifestation of SVV, it is not surprising that delayed administration of the induction immunosuppressive treatment was associated with death. Similar to the results of the studies of Cruz et al. [9] and Khan et al. [7], BVAS was also a poor predictor of ICU mortality. Indeed, a number of items in this score are a reflection much more of vasculitis activity than of an acute life-threatening manifestation of SVV. Similarly, it was not surprising that FFS was not associated with poor outcome. In the present series, as in others, cardiac symptoms or gastrointestinal involvement, two main criteria included in the FFS, were rare or not found at ICU admission [9].

### Study limitations

The present study is limited by its retrospective nature. Considering the very low incidence rate of SVV with the prespecified inclusion criteria, it would be difficult to conduct a prospective study. Nonetheless, we report a large retrospective analysis of 82 patients at 20 different centers, hence limiting center bias.

Due to the limited number of events recorded in our moderate sample size, we could not adjust for other numerous potential confounders in the multivariable analysis. Adjusting for confounders not identified as significant in this analysis could have weakened the association measured.

It is usual to report the outcome of small-vessel vasculitis at 12 and 60 months because the efficacy of the immunosuppressive therapies can be assessed only after prolonged follow-up. In the present study, we decided to report the outcome only at 90 days for the following reasons. First, the outcome at 90 days represents the specific consequences of ICU stay. Second, with a retrospective multicenter study design, data for longer-term outcomes are most often not fully available.

## Conclusions

Patients admitted to the ICU for life-threatening complications at the initial phase of SVV have an 82 % survival rate. Mortality is positively related to the intensity of organ failure. Delayed immunosuppressant use in the ICU appears to be associated with mortality. Thus, the present study sheds new light on the potential importance of a rapid approach in the treatment of these conditions.

## Key messages

- Patients admitted to the ICU for acute manifestation of small-vessel vasculitis have an 82 % survival rate.
- Even in the case of multiple organ failure, delayed administration of immunosuppressants is associated with death.

## Additional files

Additional file 1: **Outcome of patients admitted to the ICU for acute manifestation of small-vessel vasculitis.** (DOCX 23 kb)

Additional file 2: **Table 6 Comparison of 90-day survivors and nonsurvivors with regard to disease management and adverse events.** (DOCX 17 kb)

## Abbreviations

AAV: antineutrophil cytoplasmic antibody–associated vasculitis
BVAS: Birmingham Vasculitis Activity Score
CI: confidence interval
FFS: Five-Factor Score
GBM: glomerular basement membrane
GPA: granulomatosis with polyangiitis
ICD: International Classification of Diseases
ICU: intensive care unit
IQR: interquartile range
OR: odds ratio
PLEX: plasma exchange
SAPS: Simplified Acute Physiology Score
SOFA: Sequential Organ Failure Assessment
SVV: small-vessel vasculitides
