# Supplementary material for: Outcomes of patients admitted to intensive care units for acute manifestation of small-vessel vasculitis: a multicenter, retrospective study
Source: Crit Care. 2016 Jan 26;20:27. doi: 10.1186/s13054-016-1189-5 (PMC4729170; doi:10.1186/s13054-016-1189-5)
Supplement: Supplementary file 1 — Outcome of patients admitted to the ICU for acute manifestation of small-vessel vasculitis. (DOCX 23 kb) [file 13054_2016_1189_MOESM1_ESM.docx]

Additional file 1

Title : **Outcome of patients admitted in intensive care unit for acute manifestation of small vessel vasculitis**

Methodology

*Exclusion criteria*. Clinical presentation of IgA vasculitis includes purpura, arthralgia and abdominal pain, but rarely pneumo-renal syndrome. Moreover, cyclophosphamide is not known as a first line treatment in this disease [1]. Similarly, cryoglobulinemic vasculitis treatment is based on plasma exchange, corticosteroids and rituximab but not cyclophosphamide [2]. Urticarial lesions and angioedema are the main symptoms of hypocomplementemic urticarial vasculitis. First line therapy includes hydroxychloroquine or colchicine but not cyclophosphamide [3]. Consequently, because of their heterogeneous clinical presentation and management, other complex immune small vessel vasculitides (SVV) (cryoglobulinemic vasculitis, IgA vasculitis, hypocomplementemic urticarial vasculitis) were excluded from this analysis.

For each patient, the occurrence and duration of the following parameters were collected: vasopressor treatment, mechanical ventilation including non-invasive ventilation, renal replacement therapy and the length of ICU stay. Extra-Corporeal Membrane Oxygenation implantation for respiratory failure was also recorded.

Number of plasma exchanges, delay from admission to administration, duration and doses of induction immunosuppressive treatments such as corticosteroid, cyclophosphamide and eventually rituximab, were collected in both the hospitalization ward and ICU. Patients were considered with a new diagnosis of SVV if the disease was diagnosed in the previous six months. Other cases were considered as relapses of the disease. In case of relapse, chronic immunosuppressive therapy was defined if a treatment by aziathropine, cyclosporine or glucocorticoids was initiated more than six months prior to ICU admission.

*Study endpoints.* Suspicion of sepsis was considered if a new anti-infective therapy was initiated during ICU stay. Source and microorganisms as well as the occurrence of septic shock were reported.

Occurrence and source of hemorrhagic syndrome were identified. A hemorrhagic syndrome was defined by the recognition of internal or externalized bleeding requiring transfusion of red cell units. The number of red cell units and existence of hemorrhagic shock were also recorded.

Occurrence of neutropenia <1500 /mm^3^ and <500/mm^3^ following cyclophosphamide administration was recorded.

References

1. Audemard-Verger A, Pillebout E, Guillevin L, Thervet E, Terrier B: **IgA vasculitis (Henoch-Shonlein purpura) in adults: Diagnostic and therapeutic aspects**. *Autoimmun Rev* 2015, **14**:579-585.

2. Cacoub P, Comarmond C, Domont F, Savey L, Saadoun D: **Cryoglobulinemia Vasculitis**. *Am J Med* 2015.

3. Jachiet M, Flageul B, Deroux A, Le Quellec A, Maurier F, Cordoliani F, Godmer P, Abasq C, Astudillo L, Belenotti P *et al*: **The clinical spectrum and therapeutic management of hypocomplementemic urticarial vasculitis: data from a French nationwide study of fifty-seven patients**. *Arthritis Rheumatol* 2015, **67**:527-534.
