# Supplementary material for: Outcomes of patients admitted to intensive care units for acute manifestation of small-vessel vasculitis: a multicenter, retrospective study
Source: Crit Care. 2016 Jan 26;20:27. doi: 10.1186/s13054-016-1189-5 (PMC4729170; doi:10.1186/s13054-016-1189-5)
Supplement: Supplementary file 2 — Table 6 Comparison of 90-day survivors and nonsurvivors with regard to disease management and adverse events. (DOCX 17 kb) [file 13054_2016_1189_MOESM2_ESM.docx]

Table 6: Comparison between 90-day survivors and non-survivors with regard to disease management and adverse events

|  | Survivors  n=69  n (%) or median [IQR] | Non-survivors  n=13  n (%) or median [IQR] | p |
| --- | --- | --- | --- |
| Number of patients receiving glucocorticoid induction treatment | 63 (91) | 11 (84) | 0.31 |
| Number of patients receiving plasma exchanges | 52 (75) | 11 (85) | 0.72 |
| Number of patients receiving mechanical ventilation | 29 (42) | 13 (100) | <0.0001 |
| Duration of mechanical ventilation (days) | 12 [8.5-26] | 16 [9-46] | 0.1 |
| Number of patients receiving catecholamine therapy | 12 (17) | 13 (100) | <0.0001 |
| Duration of catecholamine administration (days) | 3 [2-3] | 14 [7-20] | 0.11 |
| Number of patients receiving renal replacement therapy in intensive care unit | 47 (68) | 11 (85) | 0.39 |
| Duration of renal replacement therapy in intensive care unit (days) | 13 [7-18] | 26.5 [16-49.8] | 0.075 |
| Number of patients under renal replacement therapy after intensive care unit stay | 28 (40) |  |  |
| Number of patients under renal replacement therapy prior to intensive care unit stay | 9 (13) | 2 (15) | 1 |
| Number of patients with infection | 15 (22) | 10 (77) | 0.0002 |
| Location |  |  |  |
| Urinary tract | 2 (13) | 0 (0) | 0.66 |
| Lung | 8 (53) | 7 (70) |  |
| Bacteremia | 2 (13) | 2 (20) |  |
| Others | 3 (20) | 1 (10) |  |
| Bacterial source |  |  |  |
| Gram positive | 3 (20) | 0 | 0.18 |
| Gram negative | 7 (47) | 9 (90) |  |
| Other * | 1 (7) | 0 |  |
| No pathogen identified | 4 (27) | 1 (10) |  |
| Number of patients presenting an hemorrhagic syndrome | 44 (64) | 13 (100) | 0.007 |
| Number of packed red blood cells infusions | 3.5 [0-7] | 5 [3-10] | 0.0523 |
| Number of patients presenting a septic shock | 3 (6) | 10 (85) | <0.0001 |

* virus
